# Mouse mammary tumor virus (MMTV)-like DNA sequences in the breast tumors of father, mother, and daughter

**DOI:** 10.1186/1750-9378-3-2

**Published:** 2008-02-28

**Authors:** Polly R Etkind, Alexandre FR Stewart, Peter H Wiernik

**Affiliations:** 1 Our Lady of Mercy Medical Center-Comprehensive Cancer Center, New York Medical College, Bronx, New York; 2 University of Ottawa Heart Institute, Ottawa, Ontario, Canada

## Abstract

**Background:**

The diagnosis of late onset breast cancer in a father, mother, and daughter living in the same house for decades suggested the possibility of an environmental agent as a common etiological factor. Both molecular and epidemiological data have indicated a possible role for the mouse mammary tumor virus (MMTV), the etiological agent of breast cancer in mice, in a certain percentage of human breast tumors. The aim of this study was to determine if MMTV might be involved in the breast cancer of this cluster of three family members.

**Results:**

MMTV-like envelope (*env*) and long terminal repeat (*LTR*) sequences containing the MMTV superantigen gene (*sag*) were detected in the malignant tissues of all three family members. The amplified *env *gene sequences were 98.0%–99.6% homologous to the MMTV *env *sequences found in the GR, C3H, and BR6 mouse strains. The amplified *LTR *sequences containing *sag *sequences segregated to specific branches of the MMTV phylogenetic tree and did not form a distinct branch of their own.

**Conclusion:**

The presence of MMTV-like DNA sequences in the malignant tissues of all three family members suggests the possibility of MMTV as an etiological agent. Phylogenetic data suggest that the MMTV-like DNA sequences are mouse and not human derived and that the ultimate reservoir of MMTV is most likely the mouse. Although the route by which these family members came to be infected with MMTV is unknown, the possibility exists that such infection may have resulted from a shared exposure to mice.

## Background

**Figure 1:**
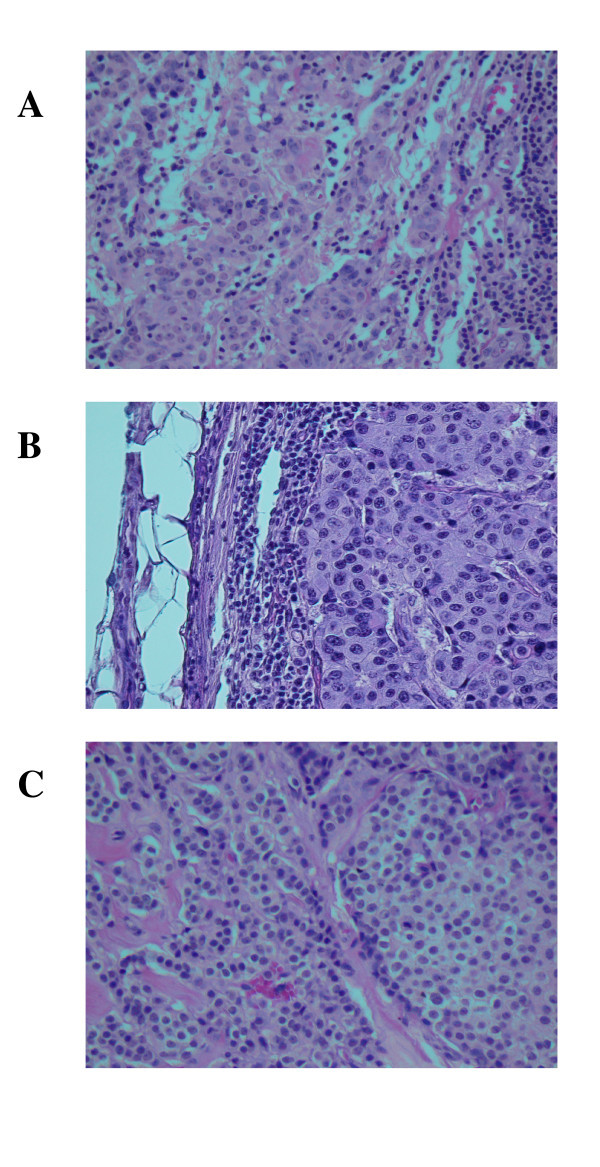
**Hematoxylin and eosin stained slides of formalin-fixed, paraffin-embedded tissue sample blocks of breast tumor tissue and metastatic breast tumor tissue in lymph node**. Panel A (father): metastatic ductal carcinoma of breast in axillary lymph node. The tumor is almost completely replacing the normal tissue in this 2-cm node. Note the rim of residual subcapsular lymphoid tissue. Panel B (mother): invasive moderately differentiated ductal carcinoma of breast. Note the prominent lymphocytic response. Panel C (daughter): invasive and in situ lobular carcinoma of breast. Only a portion of the round edge of a lobule containing lobular carcinoma in situ is seen here. Magnification is 200X.

Three members of the same family, father, mother, and daughter, were diagnosed with carcinoma of the breast with axillary nodal metastases. The father was the first to be diagnosed at the age of 79 in 1963. The mother and daughter were each diagnosed six years later in 1969 at the ages of 82 and 56 respectively. All three family members had invasive carcinoma as shown in Figure 1. Published data from five laboratories including our own have shown an association of the betaretrovirus mouse mammary tumor virus (MMTV) with a certain percentage of human breast tumors [1-5]. In addition we identified MMTV-like DNA sequences in both breast tumor tissue and non-Hodgkin's lymphoma tissue of eight patients diagnosed with both diseases and in the lymphoma tissue of three patients diagnosed with only non-Hodgkin's lymphoma [6]. We and others have not detected MMTV in normal human tissue [1,6,7]. In mice MMTV is the etiological agent responsible for the development of breast tumors as well as certain B-and T-cell lymphomas [8-10]. In this study we investigated the presence of MMTV-like DNA sequences in three family members each of whom had been diagnosed with breast cancer. We have detected the presence of both the MMTV-like envelope (*env*) and long terminal repeat (*LTR*) gene sequences in all three patients.

## Results

### Slides of tumor tissue

Figure 1A–C represents the hematoxylin and eosin stained slides from the formalin-fixed paraffin-embedded tissue sample blocks obtained from each of the three family members. Samples blocks from mother and daughter contained malignant tissue from their respective breast tumors. Sample blocks from the father were from a lymph node that contained metastatic breast cancer.

### Presence of MMTV-like *env *sequences in breast cancer

**Figure 2:**
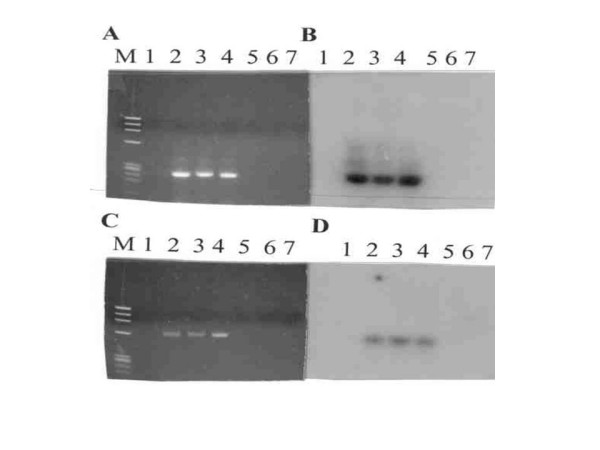
**Amplification of 250 bp of MMTV-like *env *gene and 630 bp of MMTV-like *LTR *gene by PCR.** A, Ethidium bromide stained 1% agarose gel electrophoresis of amplified MMTV-like *env *sequences; B, Southern blot [13] hybridization of A using 5' P^32 ^end-labeled 23-mer probe. Lanes 2, 3 and 4 represent the amplified DNA from the daughter, mother and father respectively. C, Ethidium bromide stained 1.8 % agarose gel electrophoresis of amplified MMTV-like *LTR *sequences; D, Southern blot [13] hybridization of C using 5' P^32 ^end-labeled 20 mer probe. Lanes 2, 3, and 4 represent amplified DNA from mother, daughter and father respectively. In Panels A-D, Lane 1 containing no template DNA and lanes 5, 6, and 7 containing normal breast tissue DNA from three separate individuals represent negative controls for sample contamination. M is the molecular weight marker ΦX174 RF DNA cut with the restriction enzyme *HaeIII*.

DNA from malignant tissue of each family member was amplified using single round PCR with primers specific for a 250 basepair (bp) region of the MMTV *env *gene and not found in any human endogenous retroviral sequences i.e HERV-Ks [1,4,11,12]. Figure 2A represents the ethidium bromide-stained agarose gel electrophoresis of an MMTV *env *primed PCR from each of the three family members and 2B is the hybridized Southern blot [13]. Lanes 2, 3, and 4 in both Figure 2A and 2B represent the amplified DNA from the daughter, mother, and father respectively. Lane 1 containing no template DNA and lanes 5, 6, and 7 containing normal breast tissue DNA from three separate individuals represent negative controls for sample contamination. Positive hybridization results with the radiolabled internal 23-mer oligonucleotide probe that contained MMTV-*env *gene sequences indicated that this MMTV-specific sequence was present in the amplified 250-bp fragment and that the bands in the agarose gel were MMTV specific.

### Presence of MMTV-like *LTR *sequences in breast cancer

DNA from the three afflicted family members was amplified by single round PCR with primers specific for a 630-bp region within the MMTV *LTR *open reading frame (ORF) that codes for the MMTV superantigen (*sag*) gene [14,15] and that is not present in HERV-K sequences [6,11,12,16]. Figure 2C represents the ethidium bromide-stained agarose gel electrophoresis of the MMTV *LTR *primed PCR of the three family members and Figure 2D represents the hybridized Southern blot [13]. Lanes 2, 3, and 4 in both Figure 2C and 2D represent amplified DNA from the mother, daughter, and father respectively. Lane 1 containing no template DNA and lanes 5, 6, and 7 containing normal breast tissue DNA from three separate individuals represent our negative controls for sample contamination. Positive hybridization with the radiolabeled internal 20-bp oligonucleotide probe that contained MMTV-*LTR *gene sequences indicated that this MMTV-specific sequence was present in the amplified 630-bp fragment and that the bands in the agarose gel were specific for MMTV *LTR *sequences.

### Cloning and sequencing of amplified MMTV-like *env *from human breast cancer

**Figure 3:**
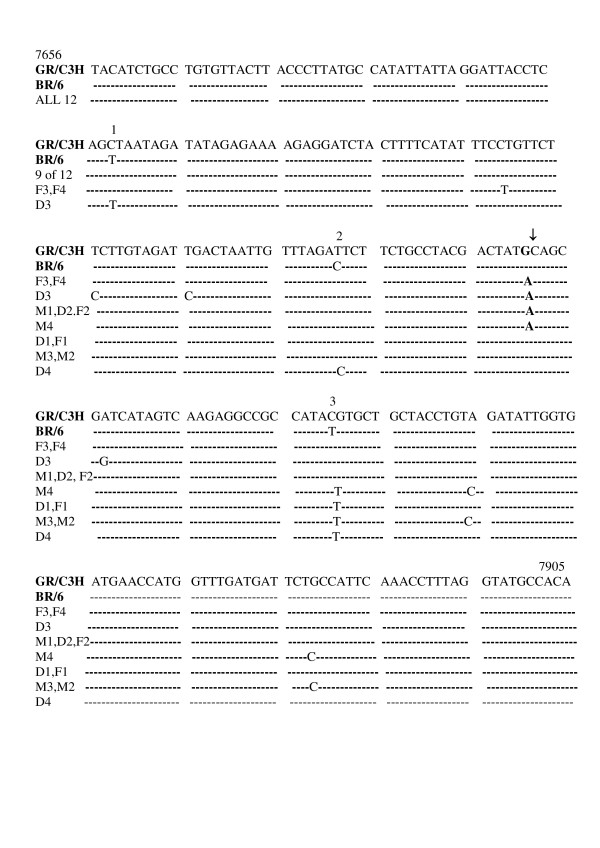
**Sequences of the 250 bp PCR MMTV-like *env *gene product amplified from the DNA of primary breast cancer tissue of mother and daughter and metastatic breast tumor tissue in lymph node of father compared with the sequences in this region of the *env *gene of GR, C3H and BR6 strains of MMTV.** The numbers 1, 2 and 3 indicate the three locations where the BR6 strain differs from the GR and C3H strains in this region of the MMTV *env *gene [17,18,19]. The **A **and ↓ indicate the location at which the identical single base change described in the text occurs. The numbers 7656 and 7905 indicate the location of the MMTV 250 bp *env *gene sequence within the MMTV genome [17]. Clones are designated as M (mother), D (daughter), and F (father) followed by a number (1–4) denoting the order in which they were cloned. - denotes the same nucleotide.

The amplified MMTV *env *gene-specific 250-bp sequences present in the primary and metastatic breast tumor tissue were cloned using the Invitrogen TOPO TA Cloning kit for Sequencing. A total of 12 clones (4 for each family member) were sequenced. The sequences of the 12 amplified fragments were shown to be 98.0% – 99.6 percent homologous to the GR, C3H and BR6 mouse strains of MMTV in this 250-bp region of the MMTV *env *gene. Figure 3 shows the comparison of the sequences of the 12 clones to the three strains of MMTV [17-19] and to each other in this region of the MMTV *env *gene.

As shown in Figure 3 the sequenced *env *clones fell into 7 classes that differed from one another. However no one *env *clone differed from any other by more than 4 base changes either within the same family member or between family members. One *env *clone each from the mother (M1), father (F2), and daughter (D2) were identical to each other. Two *env *clones from the father (F3, F4) were identical to each other, one daughter *env *clone (D1) and one father *env *clone (F1) were the same, and two *env *clones from the mother (M2, M3) were the same. Each family member however, mother (M1, M4), father (F2, F3, F4), and daughter (D2, D3), each contained *env *clones that included a single base change mutation denoted by the arrow in Figure 3. Each family member also contained *env *clones (M2, M3, F1, D1, D4) without this single base change. Previously we had identified an identical single base change in the MMTV *env *sequences that were present in a number of breast tumors and all non-Hodgkin's lymphomas [6].

### Cloning and sequencing of amplified MMTV-like *LTR *ORF sequences from human breast cancer

The amplified 630 bp MMTV-like *LTR *ORF sequences present in the primary and metastatic breast tumor tissue were cloned using the Invitrogen TOPO TA Cloning kit for Sequencing. A total of 12 clones (4 for each family member) were sequenced. The U3 region of the MMTV *LTR *contains the open reading frame (ORF) that encodes the glycoprotein superantigen (*sag*) that is present in all exogenous and endogenous MMTV viral sequences [14,15]. Although highly conserved, the MMTV sag sequences are not identical with approximately 35% of the total variation clustered at the hypervariable COOH terminus. This variation present in the COOH terminus of the MMTV *sag *gene is specific to each MMTV provirus.

**Figure 4:**
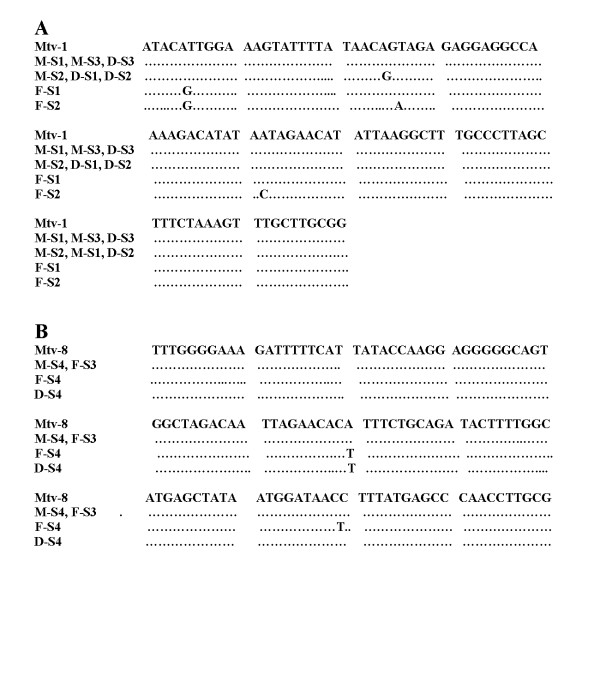
**Sequence homology of the highly variable COOH-terminal region of the MMTV *sag *gene of MMTV proviruses Mtv-1 [22,23] and Mtv-8 [20,21] to the amplified highly variable MMTV *sag *sequences cloned from the primary breast tumor tissue of the mother and daughter and metastatic breast tumor tissue in lymph node of the father.** Clones are named as M (mother), D (daughter), and F (father) followed by a dash and the letter s for *sag *and numbers (1–4) identifying the order in which they were cloned. - denotes the same nucleotide.

In the conserved regions of the *LTR *ORF all 12 clones shared 96–98 percent homology with numerous strains of MMTV and with each other. However, within the highly variable COOH terminus of the *sag *gene all of the 12 isolated clones were either identical or nearly identical to either the MMTV proviral sequence Mtv-8 [20,21] or Mtv-1 [22,23] as shown in Figure 4. Each of the three family members contained COOH-terminal *sag *sequences that were identical or nearly identical (one or two base differences) to one of these two proviral sequences.

**Figure 5:**
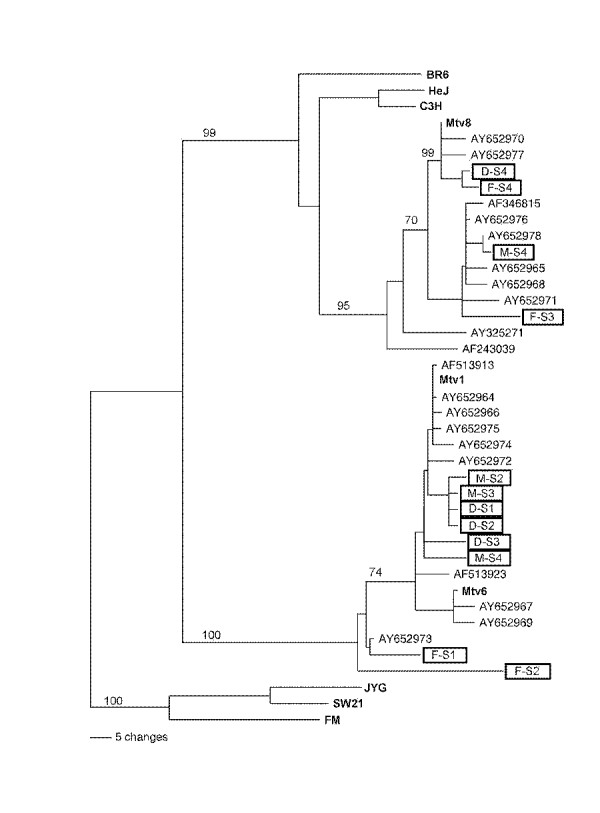
**Phylogenetic analysis of human breast tumor MMTV-like *LTR *sequences showing that the human and mouse sequences do not cluster as two distinct species.** The 12 human MMTV-like *LTR *sequences from the three family members as well as the human sequences previously isolated from human breast tumors, non-Hodgkin's lymphomas, and primary biliary cirrhosis tissue, clustered with their murine counterparts. Boxes denote *LTR *sequences from mother (M-S1-4), daughter (D-S1-4), and father (F-S1-4). Previously published human isolates AF346815, AY325271, AF243039, AY652977, AY652968, AY652964, AY652975, AY652974, AY652967, AY652969, AY652973, from human breast tumors [6,16,27], AY652970, AY652976, AY652978, AY652965, AY652971, AY652966, AY652972 from human non-Hodgkin's lymphomas [6], and AF513913, AF513923, from human primary biliary cirrhosis patients [26,27]. The mouse sequences, JYG, FM, and SW21 from Asian mice that were used to root the tree, the endogenous MMTV proviral sequences Mtv-8, Mtv-1, and Mtv-6, and the exogenous MMTV sequences BR6, HEJ, and C3H are bolded. Numbers on branches indicate percent frequencies of assortment in an individual branch after the bootstrap procedure (45) and indicate the robustness of branch assignments. Branch lengths are indicative of the number of nucleotide changes to individual branch points (see scale bar).

To analyze relationships between viral strains, phylogenetic trees have been constructed on the basis of alignments of LTR ORF sequences [24]. Phylogenetic analysis of the entire 630 bp *LTR *ORF sequences isolated from each of the three family members diagnosed with breast cancer is shown in Figure 5. All 12 of the human MMTV-like *LTR *ORF *sag *clones segregated to two branches of the MMTV phylogenetic tree, Mtv-8 [20,21] and Mtv-1 [22,23], and did not form a branch of their own. Moreover, clones isolated from the same family member segregated to these two separate branches. Interestingly, all of the prior MMTV-like *LTR *isolates from breast cancer [6,16,25], non-Hodgkin's lymphoma [5], and primary biliary cirrhosis [26,27] patients also associated with these two branches of the MMTV phylogenetic tree.

## Discussion

To our knowledge, this is the first report of breast cancer in father, mother, and daughter. We acknowledge however limitations of our study due to the quality of the DNA of the archival formalin fixed paraffin samples on which this study is based. Limitations include our inability to determine the mutation status of the BRCA1 and BRCA2 genes in these patients [28]. However the late age at which these breast tumors developed argues against their being caused by mutations in the BRCA1 or BRCA2 genes. Quantity and quality of the archival DNA also limited us in that we were able to successfully amplify only parts of two regions, the *env *and *LTR*, of the MMTV-like DNA genome and were not able to detect the presence of integrated MMTV-like viral sequences in the cellular genome.

Breast cancer in husband and wife may not be as uncommon as generally thought. At least ten couples have been previously reported since 1975 [29-33]. Russ and Scanlon [31] reporting on eleven married couples having histologically identical neoplasms, including three couples with breast cancer, noted that the clinical course of the disease tended to be similar in both husband and wife, and that husband and wife were diagnosed usually within approximately five years of each other. Both observations apply to our family. Some studies however have not shown an increased incidence of breast cancer in wives of men with that neoplasm [33-35]. However, Russ and Scanlon noted that the tumors they observed in husband and wife have been suggested either experimentally or indirectly to have viral relationships [31]. Lynch *et al *[36] suggested more than twenty years ago that a communicable agent might play a role in the clustering of certain cancers in spouses.

The MMTV-like *env *sequences that we have detected in the mother, father, and daughter of this family are 98–99.6 percent identical to the GR, C3H and BR6 mouse strains of MMTV [17-19]. Recently the Env protein of MMTV from the mouse has been shown to be capable of transforming mouse and human mammary epithelial cells *in vitro *[37]. The MMTV Env protein contains an immunoreceptor tyrosine-based activation motif (ITAM) sequence that appears to allow for its transformation ability [37]. The MMTV-like *env *gene sequences that we have isolated from the primary human breast tumor tissue and the metastatic breast tumor tissue present in a lymph node in the three family members studied in this report and in additional breast tumors previously reported and in non-Hodgkin's lymphomas contain this ITAM sequence [1,6]. It is not yet known if the MMTV-like Env protein coded for by the MMTV-like DNA sequences which we and others have detected in human breast tumors is capable of transformation. We have sequenced 4 *env *clones per family member for a total of 12 *env *clones. Seven of these 12 clones contain an identical single base substitution (Figure 3, ↓) that we have detected in a number of additional breast tumors and in all the non-Hodgkin's lymphomas we have analyzed. This base change of a G to A results in the replacement of an alanine (GCA) with a threonine (ACA) in these samples. Curiously, this single-base change occurs within the ITAM domain [37].

The 630 bp MMTV-like DNA that we have amplified using primers specific for regions of the ORF of the MMTV *LTR *codes for the superantigen (*sag*) gene of MMTV [14,15]. Although highly conserved, the MMTV sag sequences are not identical with approximately 35 percent of the total variation clustered at the hypervariable COOH terminus. This variation present at the COOH terminus of the MMTV *sag *gene is specific to each MMTV strain. Each of our cloned *sag *sequences from each family member was identical or nearly identical to either the MMTV Mtv-8 [20,21] or Mtv-1 [22,23] proviral sequence. In a previous publication [6] we have shown that the cloned *sag *sequences present in both the breast tumor tissue and non-Hodgkin's lymphoma tissue of eight patients diagnosed with both malignancies also contained *sag *sequences that were identical or nearly identical to MMTV proviral sequences Mtv-1 and Mtv-8. Similar sequences isolated from human breast tumors by others also contained *sag *sequences with identity to the Mtv-1 and Mtv-8 proviral sequences [16,25]. Also, MMTV-like *sag *sequences isolated from patients diagnosed with primary biliary cirrhosis (PBC) also contained *sag *sequences with identity to the Mtv-1 and Mtv-8 proviral sequences [26,27]. Phylogenetic studies from our laboratory and that of others [27] have shown that such sequences segregate to specific branches of the MMTV phylogenetic tree and do not form a distinct branch of their own thus arguing for their being mouse derived and not human homologues of the mouse sequence [6,27]. This study, our previously published work [6], and that of others [26,27] also indicate that more than one viral strain of MMTV-like DNA sequences may be present in the same individual.

Other works that have shown that MMTV-like DNA sequences are for the most part not found in normal cells [1-7,25] suggest that the presence of the virus in humans may result from an exogenous infection. Very recently, a virus closely related to the xenotropic murine leukemia viruses (MuLVs) has been detected in the tumor tissue of a certain population of prostate cancer patients. The viral sequence is not found in human genomic DNA thus indicating, as the authors discuss, an exogenous infection [38,39]. The suggestion that MMTV exogenous infection can occur in humans is a highly controversial topic but is becoming more plausible with two recent publications by Indik *et al *[40,41]. In these papers the authors show that MMTV can infect human cells *in vitro*, make new MMTV, and that this new MMTV can go on to infect other human cells. That the MMTV-like DNA sequences that we and others have found in human breast tumors may be mouse derived is also suggested by the findings that their *sag *sequences segregate to arms of the MMTV phylogenetic tree and do not form a separate branch of their own [6,26,27]. Additional epidemiological findings suggesting an exogenous infection from mice include the findings of Stewart *et al *[42] in which it was shown that the incidence of breast cancer was highest in countries in which the mouse strain *mus domesticus *resides, a mouse strain that carries a large number of endogenous MMTV proviruses. Curiously, a recent study from the Johns Hopkins University Schools of Medicine and Public Health has reported that airborne mouse allergen was found in 84 percent of bedrooms of inner city homes in Baltimore and that the concentration of this mouse allergen may be similar to those found in animal facilities [43]. We do not know the concentration of mouse allergen in the home of the family in this study who lived in a wealthy Baltimore neighborhood.

## Conclusion

The presence of MMTV-like DNA sequences in the malignant tissues of all three family members who lived in the same house for decades argues for the possibility of MMTV as a common environmental etiological agent. Phylogenetic data suggest that the MMTV-like sequences are mouse and not human derived and that the ultimate reservoir of MMTV is most likely the mouse. Although the route by which these three family members came to be infected with MMTV is unknown, the possibility exists that such infection may have resulted from an exposure to mice.

## Methods

### Human tissue

Formalin-fixed, paraffin-embedded tissue sample blocks of breast tumor tissue (mother and daughter) and metastatic breast tumor in lymph node (father) were obtained from the Johns Hopkins Medical Center, Baltimore, Maryland, courtesy of Drs. Elizabeth Montgomery and Arlene Forastiere, under a protocol approved by the Institutional Review Board of Our Lady of Mercy Medical Center (OLMMC). Hematoxylin and eosin stained slides of each paraffin block were made and read to determine the location of malignancy in each block. Blocks were shaved into 5 μm thick serial sections and two sections from each specimen were used for DNA extraction. DNA was isolated from the blocks using a microwave technique [44]. Normal breast tissue samples were obtained from the Pathology Department at OLMMC under a protocol approved by the Institutional Review Board of OLMMC. DNA was extracted from normal breast tissue using the QIAgen DNA Mini Kit (Qiagen Inc, Germantown, MD). To determine the quality of the isolated DNA from both paraffin blocks of tumor tissue and fresh normal breast tissue, globin primers were used in PCR and the resulting amplified products were electrophoresed in 1.8 % agarose gels.

### PCR

Conditions and primer sequences for MMTV *env *and *LTR *gene amplification were those described by Wang *et al *[4] and Liu *et al *[25] respectively. A reaction without template DNA was routinely tested to detect possible contamination of master mix components. Reactions with normal breast DNA were done to rule out contamination of tumor samples. The PCR product was analyzed by electrophoresis in 1.8 % agarose gels. ΦX174 RF DNA cut with the restriction enzyme *Hae*III was used as a marker to identify the size of the PCR products.

### Hybridization

PCR products were hybridized on Southern blots [13] under stringent hybridization conditions to either a 23-base pair (bp) probe specific for DNA sequences present in exogenous MMTV *env *sequences but not present in human endogenous retroviral sequence (HERV-K) DNA [1,4,11,12] or to a 20-bp probe specific for the MMTV *LTR *[6,16] and also not present in HERV-K DNA [11,12]. The 23-bp *env *probe, which extended from position 7822–7845 in the MMTV genome, and the 20-bp *LTR *probe, which extended from position 972–991 or 9545–9564 in the MMTV genome [17], were end-labeled with [^32^P]ATP using the T4 kinase forward reaction (Invitrogen Life Technologies, Inc., Carlsbad, CA). Stringent hybridization conditions were as described previously [1,11].

### Cloning and sequencing of PCR products

Amplified DNA products were cloned directly from the PCR tube using the TOPO TA Cloning kit for Sequencing (Invitrogen). DNA sequencing was performed by Genemed Synthesis (San Antonio, TX). The resulting sequences were compared to known published sequences and to sequences in the Genbank.

### Phylogenetic analysis

The *LTR *sequences obtained from cloned fragments after PCR amplification were compared to known exogenous and endogenous MMTV sequences in the Genbank database. *LTR *sequences were aligned using DNAssist 2.0 software and analyzed using the phylogenetic Analysis Using Parsimony (PAUP 4.0b 10) program [45] as previously described [6].

## Authors' contributions

PRE participated in the design of the study, carried out the molecular biology studies, and drafted the manuscript, AFRS carried out the phylogenetic studies and participated in the drafting of the manuscript, PHW participated in the design of the study and in the drafting of the manuscript.
